# Engineering ω-transaminase for efficient dihydroxyacetone transamination in serinol biosynthesis starting from methanol

**DOI:** 10.1016/j.synbio.2025.11.004

**Published:** 2025-11-18

**Authors:** Ya Wu, Chonghao Guo, Lizhen Deng, Derui Zhang, Yutong Bie, Yuxin He, Gen Lu, Shewei Hu, Ruiqi Zeng, Zeyang Li, Xudong Xu, Longjiang Yu

**Affiliations:** a Key Laboratory of Pesticide & Chemical Biology of Ministry of Education, Hubei Key Laboratory of Genetic Regulation and Integrative Biology, School of Life Sciences, Central China Normal University, 152 Luoyu Road, Wuhan, 430079, China; b Institute of Resource Biology and Biotechnology, Department of Biotechnology, College of Life Science and Technology, Huazhong University of Science and Technology, 1037 Luoyu Road, Wuhan, 430074, China; c Department of Biology and Food Engineering, Bozhou University, Bozhou, 236800, China

**Keywords:** C1 biomanufacturing, Serinol, Multienzyme cascade catalysis, Transaminase engineering, Epistasis analysis, Molecular simulation

## Abstract

Serinol (2-amino-1,3-propanediol) is an important pharmaceutical intermediate, but conventional chemical or microbial routes are hampered by high energy demand, product toxicity, or complex regulation. Here, we report a modular cell-free enzyme cascade, termed the methanol-to-serinol pathway (MSP), that efficiently converts methanol—a low-cost C1 feedstock—into serinol with high carbon yield. The cascade comprises two modules: Module 1 employs an alcohol oxidase and an engineered formolase to generate dihydroxyacetone (DHA), while Module 2 uses a tailored ω-transaminase for direct one-step amination. To overcome the rate-limiting DHA amination, we applied an “ALF” scanning strategy and identified a triple-mutant Cv-ωTA (Y153F/Y168F/C418F) with 6.3-fold higher specific activity than the wild type. Fitness landscape analysis revealed strong non-additive interactions, highlighting the synergistic effect of these three mutations. Molecular dynamics simulations revealed structural changes underlying the activity boost. By incorporating a pyruvate-removal system to drive the equilibrium toward product formation, the integrated cascade achieved 43.86 mM (4 g/L) serinol from 150 mM methanol in 7 h, corresponding to 87.7 % carbon yield and a productivity of 0.57 g/L/h. This work establishes a carbon-efficient route for serinol biosynthesis and provides a generalizable strategy for sustainable C1 biomanufacturing.

## 1 Introduction

**Fig. 1:**
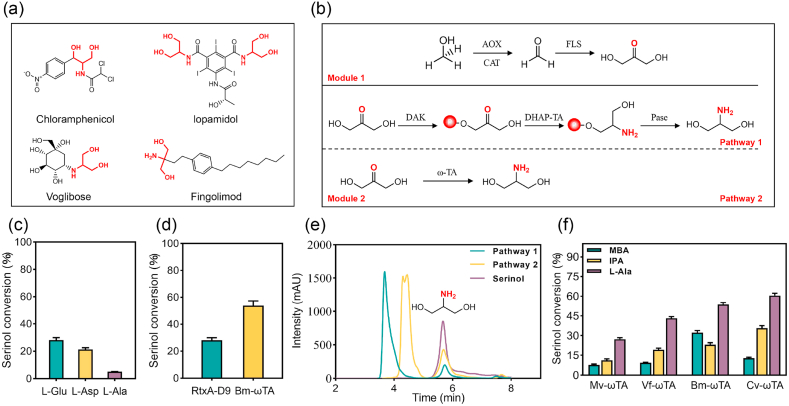
Design and validation of the modular methanol-to-serinol cascade. (a) Representative pharmaceuticals (chloramphenicol, iopamidol, voglibose, fingolimod) containing the serinol structural unit highlighted in red. (b) Schematic of the two-module pathway. AOX, alcohol oxidase; CAT, catalase; FLS, formolase; DAK, dihydroxyacetone kinase; DHAP-TA, dihydroxyacetone phosphate transaminase; Pase, phosphatase; ω-TA, ω-transaminase. (c) Effect of amino donor type (L-Glu, L-Asp, L-Ala) on DHA-to-serinol conversion in Pathway 1. (d) Comparison of serinol conversion efficiency between RtxA-D9 (Pathway 1) and Bm-ωTA (Pathway 2). (e) HPLC chromatograms of serinol produced via Pathway 1 and Pathway 2, compared with a standard. (f) Screening of ω-transaminases (Mv-ωTA, Vf-ωTA, Bm-ωTA, Cv-ωTA) with different amino donors (MBA, IPA, L-Ala) for direct DHA-to-serinol conversion.

Serinol (2-amino-1,3-propanediol) is a non-chiral aminodiol widely used as a building block in pharmaceuticals and fine chemicals. Its structural motif occurs in bioactive molecules such as the X-ray contrast agent iopamidol, the antibiotic chloramphenicol, the immunomodulator fingolimod, and the antidiabetic drug voglibose [1] (Fig. 1a). With the rise of green chemistry, demand for sustainable serinol production has intensified. Although conventional petrochemical routes have improved from ∼15 % in early processes to ∼90 % in modern processes [2], they remain energy-intensive, hazardous, and fossil-dependent [3]. These limitations highlight the urgent need for greener and innovative biocatalytic alternatives.

Microbial metabolic engineering has enabled serinol biosynthesis, offering greener and milder alternatives to chemical synthesis [4]. For example, Andreeβen et al. constructed an *E. coli* pathway using glycerol as a carbon source, in which dihydroxyacetone phosphate (DHAP) was transaminated by a phosphoserine aminotransferase (RtxA) to form phosphoserinol, then dephosphorylated to serinol [2]. This route achieved up to 3.3 g/L serinol, but RtxA tended to form insoluble inclusion bodies, limiting productivity. Luo et al. subsequently engineered RtxA for improved solubility and activity, and by using glucose as the feedstock they obtained 14.6 g/L serinol in fed-batch fermentation [1]. However, intracellular serinol accumulation proved toxic to host cells, with growth inhibition observed above ∼100 mM. This cytotoxicity imposes a ceiling on titers and requires additional derivatization or extraction steps, thereby raising cost and complicating industrial implementation [5].

In vitro multi-enzyme cascades have emerged as an attractive alternative to whole-cell systems [6]. Such cell-free approaches bypass product toxicity and metabolic regulation, enabling higher yields under optimized conditions [7]. They also allow precise control over reaction parameters (pH, temperature, cofactor levels) and facile component substitution [8,9]. Ripoll et al. demonstrated this in a preliminary validation of serinol synthesis from glycerol: glycerol was oxidized to dihydroxyacetone (DHA) by glycerol dehydrogenase and then aminated by an ω-transaminase to produce serinol [10]. Although this study reported the highest non-fermentative serinol titer to date (36 mM from 543 mM glycerol), the overall conversion was low. The transamination step was clearly the bottleneck—only 5.5 mM serinol was obtained from 100 mM DHA—highlighting the poor efficiency of the ω-transaminase with this substrate [11]. Improving transaminase activity toward DHA thus remains essential for achieving high-yielding biocatalytic serinol production.

The choice of carbon feedstock is critical for sustainable biomanufacturing. Conventional serinol biosynthesis relies on glucose or glycerol [12], but food-grade sugars compete with the food supply and lignocellulose requires costly pretreatment [13,14]. Methanol, a renewable C1 feedstock derived from CO_2_, represents a more sustainable option [15,16]. Enzyme cascades that upgrade C1 molecules into multi-carbon products have emerged as a promising paradigm. Recent breakthroughs demonstrated feasibility: Cai et al. used formolase to condense formaldehyde into DHA, which was subsequently converted into starch, sugars, and polyhydroxybutyrate [[17], [18], [19], [20]]. These advances highlight the potential of integrating methanol and formaldehyde assimilation with downstream biosynthesis to produce multi-carbon chemicals in a carbon-neutral manner.

Despite progress in serinol biosynthesis, key challenges remain. Although precursor DHA can now be synthesized from C1 feedstocks, the subsequent amination step remains the major bottleneck, preventing realization of an efficient C1-to-serinol pathway. Moreover, all previous cell-based and cell-free routes have relied on multi-carbon substrates, and no methanol-based route has been reported. Here, we establish a modular in vitro multienzyme cascade (MSP) for the green synthesis of serinol from methanol. The pathway couples formaldehyde condensation to DHA with a direct ω-transaminase–catalyzed amination step. To overcome the transamination bottleneck, we applied structure-guided engineering of the ω-transaminase, generating a triple mutant with significantly enhanced activity. Subsequent fitness landscape analysis and molecular simulations revealed the molecular basis of this activity improvement. Additionally, we introduced a pyruvate-removal module to drive the equilibrium toward serinol. This work demonstrates a scalable, highly efficient route for serinol production and contributes to the broader adoption of C1 biomanufacturing strategies.

## 2 Materials and methods

### 2.1 Assembly of the methanol-to-serinol cascade (Modules 1 and 2)

**Module 1:** The initial system consisted of 150 mM methanol, 20 μM Mr-AOX, 100 μM FLS-M9, 300 U/mL catalase (CAT), 5 mM MgCl_2_, and 1 mM TPP in 100 mM HEPES buffer (pH 7.5). The mixture was incubated at 30 °C for 5 h. To optimize enzyme loading, reactions were prepared with 150 mM methanol, 1 mM TPP, 5 mM MgCl_2_, and 100 mM HEPES buffer (pH 7.5), and incubated at 30 °C for 5 h. The concentration of Mr-AOX was varied between 20 and 40 μM with FLS-M9 fixed at 100 μM. After identifying the optimal concentration of Mr-AOX, FLS-M9 was further optimized between 100 and 180 μM. **Module 2:** (1) Phosphotransferase Assay. The reaction mixture consisted of 30 mM DHA, 0.5 mM pyridoxal-5′-phosphate (PLP), 30 mM ATP, 5 mM MgCl_2_, 150 mM amino donors (L-Glu, L-Asp, L-Ala), and 100 mM HEPES buffer (pH 7.5), and was incubated at 30 °C for 12 h. Throughout the process, the concentrations of dihydroxyacetone kinase (Kb-DAK), DHAP aminotransferase (RtxA-D9, SerC), and phosphatase (SerB) were maintained at 20 μM. (2) ω-Transaminase Assay. The reaction mixture consisted of 30 mM DHA, 0.5 mM PLP, 5 mM MgCl_2_, 150 mM amino donors (methylbenzylamine, isopropylamine, L-Ala), and 100 mM HEPES buffer (pH 7.5), and was incubated at 30 °C for 12 h. During this process, the concentrations of Cv-ωTA, Mv-ωTA, Bm-ωTA, and Vf-ωTA were maintained at 20 μM. The reaction mixture, containing 30 mM DHA, 0.5 mM PLP, 5 mM MgCl_2_, 150 mM l-alanine, and 100 mM HEPES buffer (pH 7.5), was incubated at 30 °C for 12 h. The pH of the reaction (7.0–9.0), temperature (25–45 °C), and L-Ala concentration (60–270 mM) were optimized. After each optimization step, the best-performing conditions were carried forward to the next round. This iterative process was repeated until the entire system was fully optimized.

### 2.2 Assays for serinol and intermediate compounds

Cascade reactions were conducted in 1.5 mL Eppendorf tubes in a final volume of 1 mL and terminated by adding 200 μL of 20 % trichloroacetic acid. After termination, the samples were centrifuged at 12,000 rpm for 10 min, and the supernatant was collected for analysis. Methanol, formaldehyde, DHA, and DHAP were quantified by high-performance liquid chromatography (HPLC, Agilent) equipped with an HPX-87H column (300 × 7.8 mm, Bio-Rad), with 10 mM H_2_SO_4_ as the mobile phase and a refractive index detector. The flow rate was maintained at 0.6 mL/min, and the column temperature was set to 35 °C, with an injection volume of 20 μL. Amino products (serinol, alanine, methylbenzylamine, isopropylamine) were quantified by HPLC after derivatization. The derivatization reagent was prepared by mixing 40 μg of o-phthalaldehyde, 40 μL of mercaptoethanol, and 10 mL of acetonitrile in a 25 mL amber volumetric flask, and stored at 4 °C. The derivatized samples were then analyzed by HPLC (Agilent) using a C18 column. The mobile phase consisted of Phase A (20 mM sodium acetate, prepared by dissolving 1.64 g in 1000 mL water) and Phase B (acetonitrile) in a 75:25 ratio. Detection was performed with a UV detector at 338 nm. The flow rate, column temperature, and injection volume were 0.5 mL/min, 30 °C, and 10 μL, respectively.

### 2.3 Computational design and mutant construction of Cv-ωTA

The structures of Cv-ωTA-PMP were derived from the crystal structure (PDB ID: 6S4G). Mutant protein structures were predicted using AlphaFold 3 [21]. DHA structures were optimized at the r2SCAN-3c theoretical level, and RESP2 charge fitting was performed using Multiwfn_3.8_dev [22]. The protonation states of the protein amino acids were predicted using the H++ server (http://newbiophysics.cs.vt.edu/H++/). PDBQT files for the substrate and protein were generated using AutoDock Tools. Semi-flexible molecular docking was then performed with AutoDock Vina [23,24]. Initially, the protein was docked with DHA. Each docking process was repeated three times to determine the optimal docking configuration based on affinity. Docking results were visualized using PyMOL (http://www.pymol.org) to examine protein–substrate interactions. Mutations were introduced using the wild type (WT) pET28a-Cv-ωTA plasmid as a template, with primers designed for the desired sites. PCR products were digested with *Dpn*I and transformed into *E. coli* BL21 competent cells. Sequencing confirmed the introduction of the intended mutations. Positive clones were selected and subjected to protein expression and purification.

### 2.4 Construction of pyruvate removal and recycling system

**LDH system**: The reaction mixture consisted of 100 mM DHA, 0.5 mM PLP, 5 mM MgCl_2_, 150 mM L-Ala, 5 mM NAD^+^, and 100 mM HEPES buffer (pH 7.5), and was incubated at 30 °C for 6 h. During the reaction, the concentrations of Cv-ωTA M3 and Bs-LDH were maintained at 20 μM. The system also included 200 mM sodium formate or glucose. **AlaDH system**: The reaction mixture consisted of 100 mM DHA, 0.5 mM PLP, 5 mM MgCl_2_, 150 mM L-Ala, 5 mM NAD^+^, and 100 mM HEPES buffer (pH 7.5), and was incubated at 30 °C for 6 h. During the reaction, the concentrations of Cv-ωTA M3 and Af-AlaDH were maintained at 20 μM. The system additionally contained 200 mM sodium formate or glucose. In both systems, NADH recycling was implemented using GDH and FDH, with Bm-GDH and Sc-FDH also maintained at 20 μM.

### 2.5 Optimization of module 2 reaction conditions

The initial reaction mixture included 100 mM DHA, 5 mM NAD^+^, 5 mM MgCl_2_, and 100 mM HEPES buffer (pH 7.5), along with 20 μM of the cascade enzymes. The reaction was incubated at 30 °C for 6 h. Subsequently, the following parameters were optimized: reaction pH (7.0–9.0), temperature (25–45 °C), L-Ala concentration (100–300 mM), and sodium formate concentration (100–300 mM). To optimize enzyme concentrations, we first optimized Cv-ωTA M3 concentration (5–40 μM) while keeping the other parameters unchanged. The lowest enzyme concentration that resulted in the highest serinol yield was selected as the optimal concentration for that enzyme. Based on this optimal concentration, the concentrations of the remaining enzymes, Tm-LDH and Af-AlaDH, were further optimized. NAD^+^ concentration (0–15 mM) and PLP concentration (0–1 mM) were sequentially optimized. After each step, the best-performing conditions were applied in the next round until the system was fully optimized.

### 2.6 Integration of the in vitro methanol-to-serinol cascade

In the initial stage, the reaction mixture was prepared with a final volume of 1 mL at 30 °C, containing 150 mM methanol, 1 mM TPP, 5 mM MgCl_2_, and 100 mM HEPES buffer (pH 7.5), along with the following enzymes: 300 U/mL catalase (CAT), 30 μM Mr-AOX, and 140 μM FLS-M9. The reaction was incubated at 30 °C and terminated after 4 h. The reaction mixture was then filtered through a 3 kDa microfiltration tube to remove proteins and centrifuged at 8000 rpm for 30 min. The resulting filtrate was used as the substrate for the next stage. In the second stage, a 1 mL reaction mixture was prepared containing 100 mM HEPES buffer (pH 7.5), 1 mM NAD^+^, 5 mM MgCl_2_, 0.5 mM PLP, 75 mM L-Ala, 75 mM sodium formate, and the following enzymes: 20 μM Cv-ωTA M3, 10 μM Tm-LDH, and 20 μM Sc-FDH. The reaction was incubated at 30 °C and terminated after 3 h.

### 2.7 Umbrella sampling for pre-reactive state analysis

The initial coordinates for umbrella sampling (US) were obtained from the equilibrated conformations generated during the MD simulations [25,26]. These conformations served as the starting points for three independent 10 ns NPT simulations, each used to initiate US. The reaction coordinate was defined as the distance between two heavy atoms involved in the nucleophilic addition step, specifically the nitrogen atom of PMP and the carbonyl carbon of DHA. This method was applied to compare the pre-reactive states of the WT and mutant (M3) systems. A total of 43 simulation windows were employed, with a spacing of 0.75 Å. To restrain the system along the reaction coordinate, a harmonic bias potential of 50 kcal/mol was applied. Each window was subjected to 5000 steps of energy minimization, 50 ps pre-equilibration, and a 2 ns NPT simulation. The US simulations were performed with a 1.0 fs timestep, using the same parameters as in pre-equilibration. For validation and robustness, three independent US simulations were carried out, each starting from a different equilibrated state. The Weighted Histogram Analysis Method (WHAM) was used to calculate the potential of mean force (PMF) [27].

## 3 Results

### 3.1 Design and validation of the methanol-to-serinol cascade

Serinol biosynthesis via biocatalysis requires both efficient generation and amination of DHA, a key intermediate. Whereas previous routes relied on glucose or glycerol, we proposed methanol—a one-carbon feedstock derived from CO_2_—as a more sustainable alternative. To this end, we designed a two-module cascade system termed the methanol-to-serinol pathway (MSP) (Fig. 1b). This modular design enables independent optimization of each step: Module 1 converts methanol to DHA, and Module 2 transforms DHA to serinol. In Module 1, methanol is oxidized to formaldehyde by alcohol oxidase (Mr-AOX from *Moniliophthora roreri*) and then condensed to DHA by the engineered formolase variant FLS-M9 [28], with catalase added to decompose the H_2_O_2_ byproduct. In Module 2, we evaluated two alternative routes for DHA-to-serinol conversion (Fig. 1b): a phosphorylated intermediate route (Pathway 1) and a direct one-step amination route (Pathway 2).

To determine the more efficient route for DHA-to-serinol conversion, we systematically compared two pathways. Pathway 1 mimics the natural phosphorylated intermediate route found in some microorganisms: DHA is phosphorylated to DHAP by dihydroxyacetone kinase (Kb-DAK from *Kozakia baliensis*), then transaminated to phosphoserinol either by the promiscuous phosphoserine aminotransferase RtxA-D9 from *Bradyrhizobium* or the *E. coli* serine transaminase SerC, and finally dephosphorylated by phosphoserine phosphatase (SerB) to yield serinol. Pathway 2, in contrast, simplifies the process by directly aminating DHA in a single step using ω-transaminase from *Bacillus megaterium* (Bm-ωTA), thus avoiding ATP-dependent phosphorylation and dephosphorylation. All enzymes required for both pathways were expressed in *E. coli* and purified (Fig. S1). Each module was then assembled in vitro for performance evaluation. Using 30 mM DHA as the substrate in Module 2 reactions under favorable conditions, Pathway 1 was markedly less efficient. The native *E. coli* transaminase SerC produced only trace amounts of serinol, and even replacing it with the more permissive RtxA-D9 yielded only 21.3 % conversion after 12 h, using L-Asp as the amino donor. Changing the amino donor to L-Glu increased the conversion modestly to 28.1 %. Such low yields highlight a bottleneck in this multi-step route (Fig. 1c). In contrast, Pathway 2, involving direct one-step amination, showed markedly higher efficiency: Bm-ωTA converted DHA to serinol in a single step with 54 % yield under similar conditions, using L-Ala as the amino donor, approximately double the best outcome of Pathway 1 (Fig. 1d and e). This route avoids the accumulation of phosphorylated intermediates and eliminates the need for ATP or other nucleotide cofactors, thereby improving the overall process economics and carbon efficiency. Considering these gains in both yield and operational simplicity, we selected Pathway 2 for further development.

### 3.2 Screening of ω-transaminases for DHA amination

The efficiency of the ω-TA in catalyzing the final amination is crucial for the success of the one-step DHA-to-serinol route (Pathway 2). Building on the 54 % conversion obtained with Bm*-*ωTA in initial tests, we screened additional candidate enzymes to identify an improved catalyst. Three widely studied ω-TAs—*Chromobacterium violaceum* (Cv-ωTA), *Vibrio fluvialis* (Vf-ωTA), and *Mycobacterium vanbaalenii* (Mv-ωTA)—were evaluated, with Bm-ωTA serving as a benchmark. Each enzyme was assayed for its ability to aminate DHA, and different amino donors were also tested to help overcome thermodynamic equilibrium constraints. Prior studies have shown that donor choice can shift equilibrium toward the desired amine [29]. Accordingly, three common donors were compared: (S)-α-methylbenzylamine (MBA), isopropylamine (IPA), and L-Ala. As shown in Fig. 1f, all ω-TAs gave substantially higher serinol conversion with L-Ala than with IPA or MBA, confirming L-Ala as the most effective donor. Under these conditions, Cv-ωTA was the most effective, converting ∼60 % of DHA to serinol, whereas Mv-ωTA achieved only 27.3 %. Therefore, Cv-ωTA with L-Ala proved to be the optimal catalyst–donor combination, outperforming Bm-ωTA. We therefore selected Cv-ωTA as the lead enzyme for further optimization in the cascade.

**Fig. 2:**
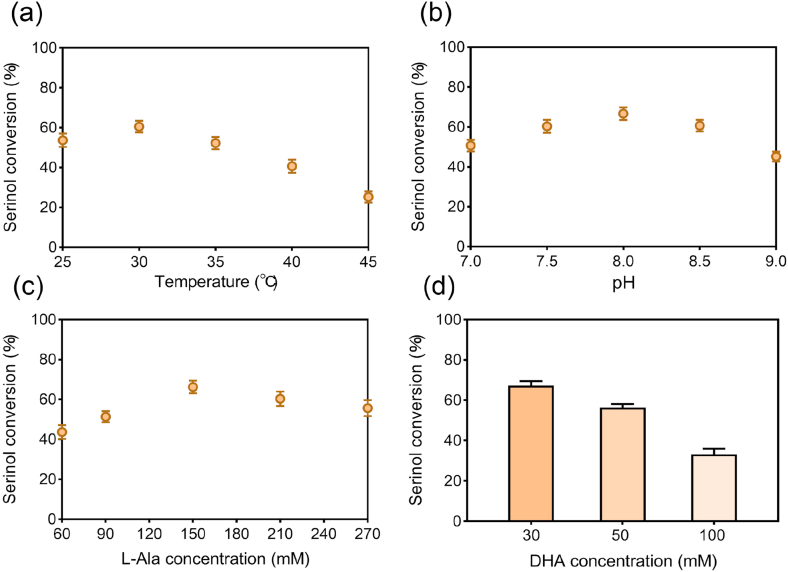
Optimization of Cv-ωTA reaction conditions for DHA-to-serinol conversion. (a) Optimization of reaction temperature (25–45 °C). (b) Optimization of reaction pH (pH 7.0–9.0). (c) Optimization of L-Ala concentration (60–270 mM). (d) Serinol conversion efficiency of Cv-ωTA at different DHA substrate concentrations.

Having identified Cv-ωTA as the superior transaminase, we optimized its reaction conditions to maximize efficiency. Temperature and pH profiling indicated optimal activity at 30 °C and pH 8, which were adopted for subsequent experiments. We next assessed the influence of amino donor concentration (Fig. 2a–c). Increasing the L-Ala concentration generally shifted the equilibrium toward serinol, with the highest conversion observed at 150 mM (five equivalents of DHA). At higher concentrations, conversion decreased, as has been previously observed for other ω-transaminases, where excess amino donors hindered the reaction efficiency [30]. Thus, 150 mM L-Ala was chosen as the optimal donor concentration. Finally, we examined Cv-ωTA's performance at elevated DHA concentrations to evaluate its suitability for high-titer production (Fig. 2d). At 50 mM DHA, 56.4 % of substrate was converted to serinol, but efficiency declined sharply at higher concentrations. At 100 mM DHA, only 33.1 % conversion was observed, despite doubling the enzyme loading to 40 μM. This decline indicates that Cv-ωTA is inherently limited under substrate concentrations relevant to industrial-scale production. Neither increasing enzyme loading nor further parameter optimization alleviated this limitation, confirming that the transaminase step constitutes the major bottleneck for achieving high serinol titers. In light of this, we concluded that protein engineering of Cv-ωTA would be necessary to boost its catalytic efficiency and enable efficient conversion at high substrate concentrations.

### 3.3 Rational engineering of Cv-ωTA using the ALF scanning strategy

**Fig. 3:**
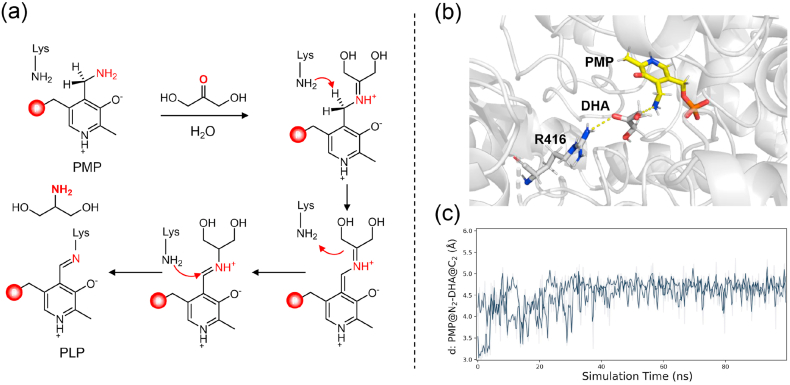
Mechanistic basis for the poor activity of Cv-ωTA toward DHA. (a) Ping–pong bi–bi mechanism of Cv-ωTA catalysis in second half-reaction, where PMP reacts with DHA to form serinol. (b) Representative conformation from the MD simulation of DHA and PMP in the Cv-ωTA active site, showing weak binding with only one hydrogen bond involving residue R416. (c) MD simulation of the PMP–DHA complex, showing a long average distance (>4.5 Å) between PMP (N_2_) and DHA (C_2_).

To overcome the catalytic limitations of Cv-ωTA with DHA, we pursued a rational engineering strategy guided by mechanistic insight. Cv-ωTA is a typical PLP-dependent transaminase that operates via a ping-pong bi–bi mechanism, where serinol formation occurs in the second half-reaction [31]. This involves the deamination of the donor (L-Ala) to pyruvate, followed by the conversion of PLP to PMP, after which the PMP cofactor transfers the amino group to DHA (Fig. 3a). We hypothesized that the enzyme's poor performance at high DHA concentrations arises from suboptimal substrate binding or positioning in the active site during this step. To test this, DHA was docked into the Cv-ωTA active site and molecular dynamics (MD) simulations (2 × 100 ns) were performed on the Cv-ωTA–PMP–DHA complex. The simulations revealed that DHA bound loosely in the native Cv-ωTA. Despite two hydroxyl groups, it displayed high conformational flexibility and maintained only a single stable hydrogen bond with active-site residues (R416) (Fig. 3b; Fig. S2). Moreover, the critical distance between the PMP amino group (N_2_) and the DHA carbonyl carbon (C_2_) fluctuated above 4.5 Å, much longer than required for efficient nucleophilic attack [32] (Fig. 3c). These observations suggest that the native active-site cavity is too spacious and not optimally shaped to hold DHA in a productive conformation, leading to inefficient amination.

Guided by these insights, we planned to redesign the Cv-ωTA active site to better accommodate DHA. Through computational modeling, we identified ten key amino acid residues (positions 22, 60, 88, 153, 166, 168, 231, 416, 417, and 418) in the substrate pocket that play a crucial role in determining the pocket's geometry and its interactions with DHA. The WT pocket is predominantly hydrophobic and relatively spacious for a small substrate like DHA, we introduced mutations to systematically modulate pocket size and hydrophobic contacts. Specifically, we constructed a focused mutagenesis library based on an “ALF” scanning strategy, explicitly named for the amino acids used: Alanine (small), Leucine (medium), and Phenylalanine (large). This approach employs this set of hydrophobic residues as “molecular rulers” to systematically vary side-chain bulk, thereby precisely tuning the active-site geometry and hydrophobic contacts to optimize accommodation for the small substrate DHA [33].

**Fig. 4:**
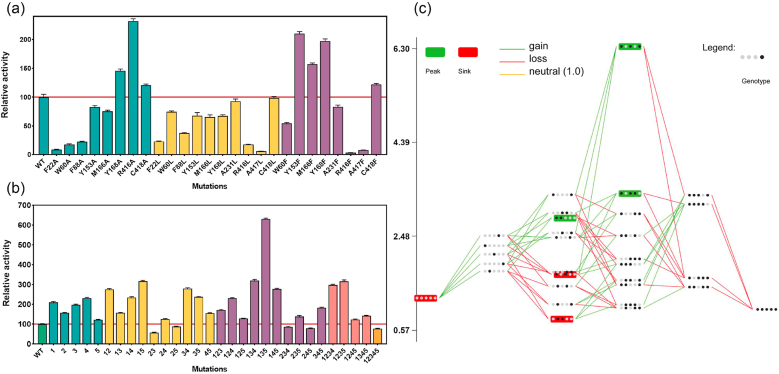
Activity and evolutionary pathway analysis of Cv-ωTA variants. (a) Relative activities of single-point mutants toward DHA compared with WT. (b) Relative activities of multi-point mutants compared with WT. The mutations include: Y153F (1), M166F (2), Y168F (3), R416A (4), and C418F (5). (c) Nodes represent different mutational combinations, and edges indicate evolutionary trajectories. Green and red lines denote gain- and loss-of-function paths, respectively. These illustrate the interactions and epistatic relationships among the substitutions.

A total of 26 single mutants were generated by introducing point mutations at various positions, and their activities were assayed. Screening of these mutants identified five substitution sites that significantly enhanced activity toward DHA (Fig. 4a). The most effective mutation was R416A, which increased activity approximately 2.3-fold relative to the WT. Additionally, four phenylalanine substitutions—Y153F, M166F, Y168F, and C418F—also improved the enzymatic activity. However, several mutations in key residues led to nearly complete loss of activity (F22A, R416F, A417L, A417F), highlighting the critical importance of these residues in maintaining enzyme function. These results validate our design rationale: by tuning the size and hydrophobicity of hotspot residues, we enhanced substrate accommodation and optimized the active site for better catalytic efficiency. Substitutions with Phe or Ala improved steric complementarity and reduced polar interference, thereby stabilizing DHA in a more productive binding mode.

### 3.4 Fitness landscape analysis and characterization of the optimal mutant

Having identified five beneficial single-site mutations in Cv-ωTA (Y153F, M166F, Y168F, R416A, and C418F), we constructed a comprehensive fitness landscape encompassing all 32 possible combinations to investigate how these mutations affect each other and determine the optimal mutational trajectories. Enzyme activity served as the fitness proxy. The results revealed strong non-additive interactions (high-order epistasis) among the mutations (Fig. 4b). Notably, the full quintuple mutant retained only 77 % of WT activity, indicating that simply combining all substitutions reduced performance [34]. In contrast, a specific triple mutant called M3, comprising Y153F, Y168F, and C418F, was the most potent variant, with specific activity 6.3-fold that of the WT. This enhancement far exceeded the expected sum of individual effects, demonstrating strong positive epistasis among the three substitutions [35].

Analysis of mutational combinations revealed that only certain evolutionary paths avoided performance cliffs caused by harmful intermediates. The mutations Y153F, Y168F, and C418F together formed a synergistic core critical for significant activity gains. The pair Y153F + Y168F alone showed less improvement than expected, as these two mutations interfered with each other. However, the introduction of C418F restored activity, resulting in a strong compensatory effect. To gain deeper insight into the epistatic interactions underlying M3, we analyzed evolutionary trajectories involving its key mutations using MAGELLAN [36]. This analysis confirmed complex, non-additive interactions (Fig. 4c; Fig. S3) . In particular, Y153F and Y168F exhibited reciprocal sign epistasis (i.e., where each mutation is beneficial individually, but their combination is deleterious) unless combined with C418F. Mapping the full landscape also showed that the order in which mutations were acquired significantly impacted the results. Many paths toward the quintuple mutant encountered low-activity intermediate mutants, hindering further progression. Only a limited subset of trajectories avoided such drop-offs. The success of M3 underscores the importance of strategic, stepwise accumulation of beneficial mutations, rather than indiscriminate combination.

**Fig. 5:**
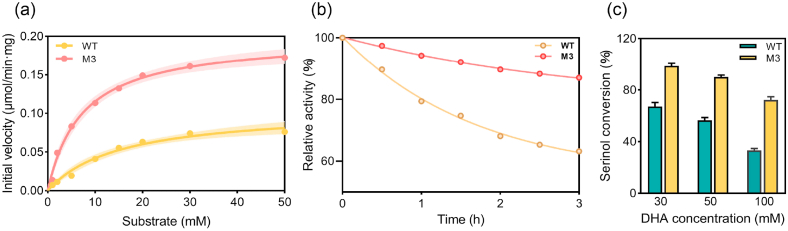
Comparative performance of WT and M3 (the triple mutant). (a) Kinetic curves of WT and M3 with DHA as substrate. (b) Thermal stability profiles at 50 °C over 3 h. (c) Serinol conversion by WT and M3 at different DHA concentrations.

To validate the functional advantages of the optimized variant, we compared the kinetic properties and thermal stability of WT and the M3 mutant (Fig. 5). M3 exhibited marked improvements in both catalytic efficiency and thermal stability. Kinetic analysis showed a significantly reduced *K*_m_ and an increased *k*_cat_, resulting in a *k*_cat_/*K*_m_ ratio 4.2-fold that of the WT (Table S1). This demonstrates that M3 not only enhances substrate affinity but also markedly improves catalytic efficiency. Thermal stability assays further supported this, showing 87 % residual activity for M3 after incubation at 50 °C for 3 h, compared to 63 % for the WT. We further evaluated the conversion performance of M3 across different DHA concentrations (Fig. 5c). At 30 mM DHA, conversion increased from 66.3 % (WT) to 95.7 % with M3. At 50 mM, M3 still achieved a high conversion (90.1 %), but at 100 mM, the yield declined to 72.3 %. These results indicate that M3 substantially improves catalytic performance at moderate substrate levels. The decline at higher DHA concentrations is attributable to the reversibility of the transamination reaction, where accumulation of substrate and byproducts shifts the equilibrium away from product formation.

### 3.5 Structural basis and mechanistic analysis of M3 activity enhancement

**Fig. 6:**
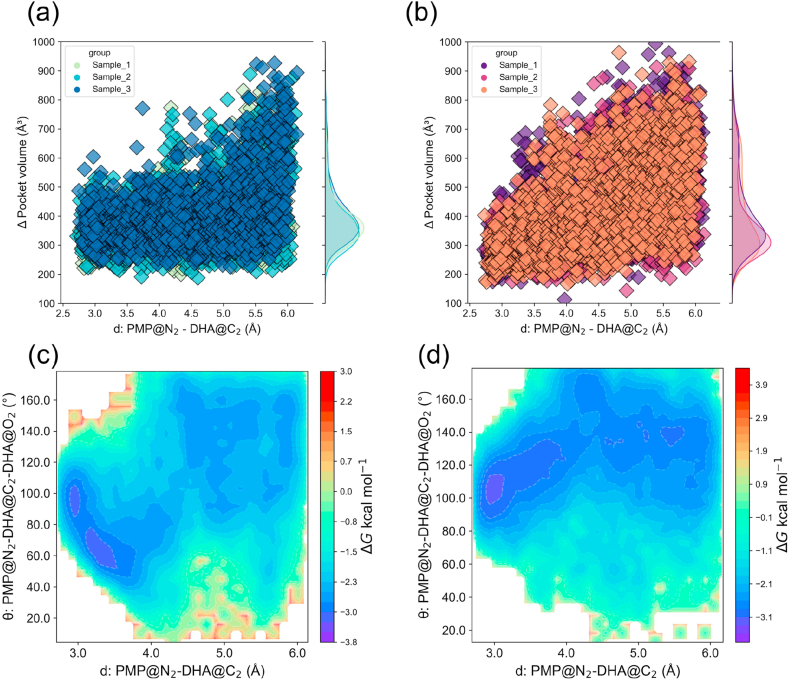
Active-site pocket volume and free energy landscape of WT and M3. Correlation between the active-site pocket volume and the distance between the nitrogen atom of PMP (N_2_) and the carbonyl carbon of DHA (C_2_) in WT (a) and M3 (b). Free energy landscapes showing the relationship between the PMP (N_2_)–DHA (C_2_) distance and the Bürgi–Dunitz angle, both of which are critical for optimal nucleophilic attack alignment, in WT (c) and M3 (d).

To elucidate why M3 exhibits superior activity in DHA transamination, we analyzed its structural and mechanistic features. The structure of M3 was predicted using AlphaFold 3, and DHA was docked into the active site. Two independent 100 ns molecular dynamics simulations were then performed. Compared to the WT, the M3 active site formed a denser interaction network with the substrate (Fig. S4b). In these simulations, M3 consistently maintained 3–4 hydrogen bonds with DHA, whereas the WT maintained only 1–2 hydrogen bonds. The nucleophilic attack distance was also markedly reduced [37,38] (Fig. S4c). Further structural analysis revealed the structural basis of M3's enhanced catalytic activity. Trajectory data showed that the average cavity volume of the M3 active site was reduced to ∼315 Å^3^, compared with ∼353 Å^3^ in the WT (Fig. 6a and b), representing an 11 % decrease [39]. This contraction reshaped the active-site geometry, restricting random substrate motion and stabilizing its bound conformation [40]. In addition, the structural changes reduced solvent interference and dampened DHA fluctuations through hydrophobic interactions, thereby optimizing substrate binding.

To quantify the impact of these structural differences on the reaction coordinate, we performed umbrella sampling simulations to profile the free energy landscape of the bond-forming step. Two geometric parameters were examined: (1) the distance between the nitrogen atom of PMP (N_2_) and the carbonyl carbon of DHA (C_2_), and (2) the Bürgi–Dunitz angle formed by the nitrogen atom of PMP (N_2_), the carbonyl carbon of DHA (C_2_), and the carbonyl oxygen of DHA (O_2_), which ideally approaches ∼109° for optimal nucleophilic alignment [41]. The free energy profiles (Fig. 6c, d, Fig. S5) revealed that M3 stabilizes a more favorable reactive conformation than the WT, with a distinct free-energy minimum at a shorter PMP (N_2_)–DHA (C_2_) distance. In addition, the M3 active site stabilized a near-ideal Bürgi–Dunitz angle (∼109°), whereas the WT exhibited less optimal alignment [42]. In summary, the M3 possesses a geometrically and chemically refined active site, featuring enhanced hydrogen bonding, optimized spatial constraints, and near-ideal substrate alignment for nucleophilic attack. These mechanistic features explain the observed catalytic enhancement of M3 and align with the improvements achieved through enzyme engineering and evolutionary pathway analyses.

### 3.6 Pyruvate removal and recycling strategies to enhance serinol production

**Fig. 7:**
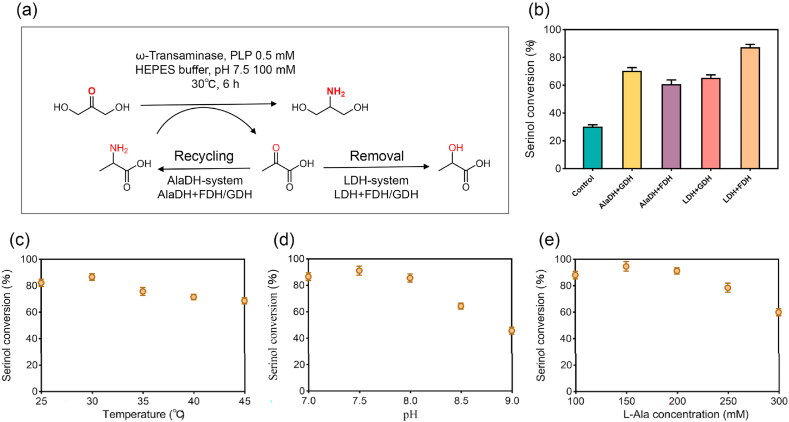
Pyruvate removal/recycling strategies and optimization of Module 2. (a) Schematic of pyruvate removal (LDH + FDH/GDH) and recycling (AlaDH + FDH/GDH) systems coupled to ω-transaminase. (b) Comparison of serinol conversion with different removal and recycling systems. (c) Optimization of reaction temperature (25–45 °C). (d) Optimization of reaction pH (7.0–9.0). (e) Optimization of L-Ala concentration (100–300 mM).

In alanine-mediated transamination, pyruvate accumulates as a byproduct and accepts the amino group from the released serinol to regenerate alanine. This shift in equilibrium toward alanine inhibits serinol formation. Although enzyme engineering alleviated the kinetic bottleneck, overall conversion remained thermodynamically constrained. To overcome this limitation, we introduced auxiliary enzyme modules to continuously remove pyruvate, thereby pulling the transamination equilibrium toward product formation [43] (Fig. 7a). Prior studies showed that diverting pyruvate to other metabolites, such as lactate or alanine, can favor product formation in ω-TA reactions [44,45]. Inspired by these reports, we evaluated pyruvate removal/recycling modules: lactate dehydrogenase (LDH) to reduce pyruvate to lactate, and alanine dehydrogenase (AlaDH) to reductively aminate pyruvate back to L-Ala [46]. In each case, a cofactor regeneration enzyme (formate dehydrogenase, FDH, or glucose dehydrogenase, GDH) supplied the required NADH. This four-enzyme coupling strategy was designed to drive the ω-TA equilibrium toward serinol by continually removing inhibitory pyruvate. Enzyme activity assays identified the optimal combinations for cascade assembly (Table S2). As shown in Fig. 7b, all pyruvate removal/recycling systems substantially improved serinol yield. Under conditions of 100 mM DHA and 1.5 equivalents of L-Ala after 6 h, LDH + FDH achieved 87.4 % conversion, whereas AlaDH + GDH reached 70.4 %. These results confirm that eliminating or recycling pyruvate effectively shifts equilibrium toward product formation. The LDH/FDH system was the most effective at accelerating serinol synthesis, whereas the AlaDH route, by regenerating the amino donor, provided an additional practical means to drive reactions toward high conversion.

### 3.7 Optimization of Module 2 for high-yield serinol production

**Fig. 8:**
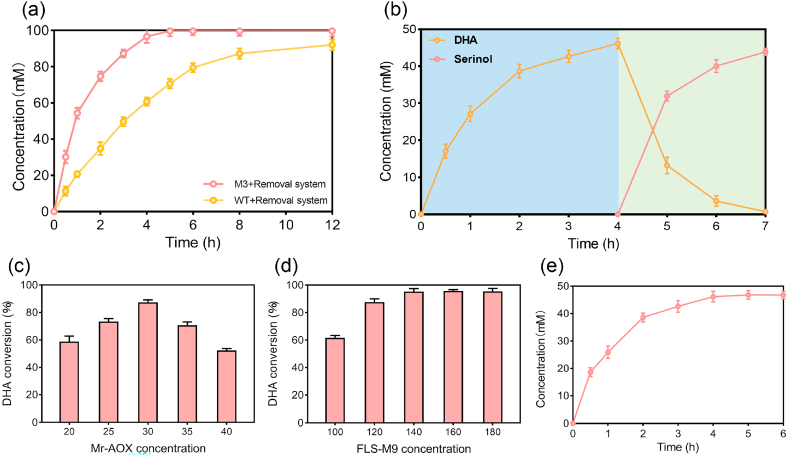
Time-course analysis of serinol production and optimization of Module 1 for DHA formation. (a) Time course of serinol production from DHA using WT with pyruvate removal system, and M3 with pyruvate removal system over 12 h. (b) Two-step methanol-to-serinol cascade showing DHA accumulation in Module 1 (blue-shaded area) and subsequent serinol production in Module 2 (green-shaded area). (c) Optimization of Mr-AOX concentration (20–40 μM). (d) Optimization of FLS-M9 concentration (100–180 μM). (e) Time course of DHA accumulation derived from 150 mM methanol over 6 h.

Having established an enhanced Module 2 with the pyruvate-removal strategy and engineered ω-TA, we optimized the reaction conditions to maximize serinol yield. Temperature and pH screening confirmed 30 °C as optimal, while the best pH shifted slightly from 8.0 to 7.5. We then optimized substrate and cofactor concentrations (Fig. 7c and d), achieving the highest performance with 100 mM DHA as the substrate, 150 mM L-Ala as the amino donor, and 150 mM sodium formate (Fig. 7e). This balanced combination enabled complete cycling without the large donor excess (5–10 equivalents) typically required in single-step transaminations, thus reducing L-Ala consumption and associated costs. We further minimized enzyme loading by varying the concentrations of ω-TA and auxiliary enzymes. The optimal combination consisted of 20 μM M3, 10 μM Tm-LDH, and 20 μM Sc-FDH, along with the cofactors 0.25 mM PLP and 1 mM NAD^+^ (Fig. S6). This minimal enzyme set enabled near-quantitative conversion of DHA to serinol, reaching 99.7 % of the theoretical yield from 100 mM DHA. Importantly, under the optimized setup the WT required 12 h to reach 92.1 mM serinol, whereas M3 produced 96 mM in just 4 h—a three-fold shorter reaction time (Fig. 8a). Together, the optimized conditions and the superior M3 catalyst enabled Module 2 to produce serinol both rapidly and at high yield, laying a robust foundation for scale-up and integration with Module 1.

### 3.8 Optimization of Module 1 for high-yield DHA production

We next optimized Module 1, the methanol-to-DHA conversion, to maximize upstream DHA supply. Our previous work yielded a highly active formolase mutant (FLS-M9) for condensing formaldehyde and glycolaldehyde into DHA [28], which we paired with methanol oxidase (Mr-AOX) to construct the Module 1 cascade. Starting with 150 mM methanol, we first optimized Mr-AOX concentration. Increasing the enzyme from 20 μM to 30 μM markedly improved DHA formation, boosting conversion to 87.4 % of the theoretical maximum (Fig. 8c). However, further raising Mr-AOX to 35 μM reduced DHA yield, likely due to over-oxidation of methanol or intermediate formaldehyde to formic acid, an undesired byproduct [47]. We then optimized the FLS-M9 catalyst loading (Fig. 8d). DHA yield increased with FLS-M9 concentration and plateaued around 140 μM, with no further improvement at higher levels. Under the combined optimal conditions (30 μM Mr-AOX and 140 μM FLS-M9), Module 1 produced DHA efficiently. As shown by time-course measurements (Fig. 8e), DHA accumulated rapidly and reached 46.2 mM after 4 h, corresponding to 92.3 % of the theoretical yield from 150 mM methanol. These optimizations underscore the effectiveness of the methanol-to-DHA module and provide a solid foundation for the full methanol-to-serinol cascade.

### 3.9 Integration of Modules 1 and 2 for methanol-to-serinol conversion

Finally, we integrated the optimized Module 1 and Module 2 into a complete cascade for serinol synthesis from methanol. Initial trials showed that a stepwise configuration was required for high efficiency, rather than combining all enzymes in a single batch. This is because residual methanol and its intermediate formaldehyde from Module 1 inhibits Module 2 enzymes; for example, high methanol concentrations can inactivate transaminases. Running the two modules sequentially allowed each stage to operate under optimal conditions while avoided inhibitory cross-talk. Experimentally, 150 mM methanol was first converted to DHA in Module 1 under the optimized conditions (4 h). The resulting reaction mixture was then ultrafiltered to remove Module 1 enzymes (Mr-AOX and FLS-M9), yielding a clarified DHA solution. This intermediate served as the substrate for Module 2. In this stage, the refined enzyme cocktail (M3, LDH, FDH, along with the required cofactors) was added, and transamination proceeded for an additional 3 h. The two-step cascade achieved an overall serinol titer of 43.86 mM, equivalent to 4 g/L, from 150 mM methanol (Fig. 8b). This corresponded to a carbon yield of 87.7 % and a volumetric productivity of 0.57 g/L/h, based on the total 7 h reaction time.

## 4 Discussion

In this study, we constructed and optimized a modular in vitro multienzyme cascade (MSP) capable of efficiently converting methanol into serinol. By integrating rational pathway design, targeted engineering of the rate-limiting enzyme, mechanistic analysis, and process optimization, this work provides a more sustainable alternative to conventional production methods and advances the field of C1 biomanufacturing. In this two-module cascade pathway, methanol was first oxidized to DHA, followed by direct amination to serinol in a single transamination step. This in vitro, ATP-independent design bypasses the phosphorylation steps required in microbial routes, thereby avoiding the cytotoxicity caused by intracellular serinol accumulation. Furthermore, the use of methanol, a renewable C1 resource that does not compete with food supplies, offers sustainability advantages compared with traditional multi-carbon feedstocks such as glucose [48]. The modularity of the cell-free system enables independent optimization of each reaction stage and facilitates integration of a pyruvate-removal module to overcome the equilibrium limitations of the transamination step. Owing to these features, we achieved 43.86 mM serinol (4 g/L) from 150 mM methanol with a carbon yield of 87.7 %. This carbon yield is 13.3-fold higher than that of the best previously reported cell-free multienzyme system (∼6.6 %, Ripoll et al.), with a volumetric productivity of 0.57 g/L/h, nearly fivefold that of the best fed-batch fermentation record (0.122 g/L/h, Luo et al.) [2,10]. Collectively, this work establishes a highly carbon-efficient route for green serinol synthesis and provides a robust example for sustainable C1 biomanufacturing.

This study also addressed the bottleneck of poor catalytic efficiency of ω-transaminases toward small substrates such as DHA [49]. Recent engineering efforts in the field have largely focused on two themes: (i) enhancing catalytic performance (e.g., improving activity for bulkier aromatic substrates by remodeling active sites), or (ii) improving overall enzyme robustness and process tolerance (e.g., toward furfural or in co-solvents) [[50], [51], [52], [53], [54]]. In contrast to the common strategy of pocket expansion, our work applied the ALF-scanning strategy to address a distinct challenge: not to enlarge the pocket, but to systematically remodel and constrict it for the atypical, small substrate DHA. Using this rational design approach, we obtained a triple mutant, M3, that exhibited a specific activity 6.3-fold higher than that of the wild type, demonstrating the general applicability of ALF scanning for tailoring enzymes to synthesize small-molecule amines. Fitness landscape analysis further revealed strong non-additive epistatic effects: the three mutations in M3 did not act additively. For example, the double mutant Y153F/Y168F exhibited a negative epistatic interaction. However, the addition of C418F compensated for this and resulted in a synergistic effect, allowing M3 to outperform even more complex mutational combinations. These results illustrate evolutionary accessibility, showing mutational order was critical to avoid low-activity intermediates (Fig. 4c), and caution against the flawed assumption of additivity. Molecular dynamics simulations further elucidated the structural basis of this synergy, clarifying the conformational changes underlying the activity enhancement. These results underscore the importance of mapping fitness landscapes to guide protein engineering, allowing for the identification of beneficial combinations while avoiding deleterious interactions, thereby enabling more efficient enzyme optimization.

Notably, during the course of this work, Fan et al. reported an enzymatic cascade for serinol synthesis starting from formaldehyde [55]. Their work focused on the upstream synthesis of DHA, whereas our study addressed the downstream bottleneck of DHA amination. The two approaches are thus highly complementary in the development of a complete C1-based serinol pathway. A key feature of our process is the direct use of methanol as the C1 feedstock, which is readily available and offers advantages in operational handling. Furthermore, our optimized transamination reaction achieved high efficiency with only 1.5 equivalents of amino donor, a significant improvement over conventional ω-transaminase systems that often require a large excess (5–10 equivalents) to drive equilibrium [56]. This combined strategy significantly improves the overall atom economy and environmental sustainability.

Despite these achievements, several challenges remain to be addressed before this modular in vitro multienzyme system can be industrially applied. For instance, the cost and long-term operational stability of enzymes, particularly for high-load catalysts like FLS-M9 (140 μM), may hinder large-scale implementation. Looking ahead, emerging protein engineering tools such as machine-learning–guided directed evolution and protein language models could be employed to further enhance ω-transaminase performance, as well as to improve the solubility and robustness of key cascade components like FLS-M9 [57]. Moreover, integrating this multienzyme cascade into continuous-flow bioreactors and conducting scale-up studies will be essential for industrial application [58]. The strategies developed in this work can also be extended to the synthesis of other amino alcohols and related compounds from C1 feedstocks, thereby providing new avenues toward a circular bioeconomy.

## CRediT authorship contribution statement

**Ya Wu:** Writing – original draft, Visualization, Methodology, Investigation, Formal analysis, Data curation, Conceptualization. **Chonghao Guo:** Resources, Methodology, Formal analysis. **Lizhen Deng:** Methodology, Investigation. **Derui Zhang:** Supervision, Resources. **Yutong Bie:** Supervision, Resources. **Yuxin He:** Supervision, Resources. **Gen Lu:** Software. **Shewei Hu:** Resources. **Ruiqi Zeng:** Methodology. **Zeyang Li:** Methodology. **Xudong Xu:** Writing – review & editing, Resources, Funding acquisition. **Longjiang Yu:** Writing – review & editing, Funding acquisition, Conceptualization.

## Funding sources

This work was financially supported by the 10.13039/501100012226Fundamental Research Funds for the Central Universities (No.CCNU24JCPT018) and HUST (NO.YCJJ20242225), the 10.13039/501100012166National Key R&D Program of China (2022YFC2106000), and the 10.13039/501100018583Wuhan Science and Technology Key Projects (2023020302020708).

## Declaration of competing interest

The authors declare that they have no known competing financial interests or personal relationships that could have appeared to influence the work reported in this paper.
