# Supplementary material for: Engineering ω-transaminase for efficient dihydroxyacetone transamination in serinol biosynthesis starting from methanol
Source: Synth Syst Biotechnol. 2025 Nov 18;12:71–81. doi: 10.1016/j.synbio.2025.11.004 (PMC12666128; doi:10.1016/j.synbio.2025.11.004)
Supplement: Multimedia component 1 [file mmc1.doc]

Engineering ω-transaminase for efficient dihydroxyacetone transamination in serinol biosynthesis starting from methanol

Ya Wu^a^, Chonghao Guo^b^, Lizhen Deng^b^, Derui Zhang^b^, Yutong Bie^b^, Yuxin He^b^, Gen Lu^b^, Shewei Hu^c^, Ruiqi Zeng^b^, Zeyang Li^b^, Xudong Xu^a*^, Longjiang Yu^b*^

^a^ Key Laboratory of Pesticide & Chemical Biology of Ministry of Education, Hubei Key Laboratory of Genetic Regulation and Integrative Biology, School of Life Sciences, Central China Normal University, 152 Luoyu Road, Wuhan 430079, China

^b^ Institute of Resource Biology and Biotechnology, Department of Biotechnology, College of Life Science and Technology, Huazhong University of Science and Technology, 1037 Luoyu Road, Wuhan 430074, China

^c^ Department of Biology and Food Engineering, Bozhou University, Bozhou 236800 , China

***Corresponding Authors:**

^*^Longjiang Yu: [yulongjiang@hust.edu.cn](mailto:yulongjiang@hust.edu.cn)

^*^Xudong Xu: xuxudong@ccnu.edu.cn

**Table of Contents**

[Materials and methods 3](#__RefHeading___Toc10623)

Fig. S1 SDS-PAGE analysis of recombinant enzymes involved in the methanol-to-serinol cascade 7

Fig. S2 MD simulations of Cv-ωTA (WT) with DHA 7

Fig. S3 Epistasis analysis of key Cv-ωTA mutations (Y153F, Y168F, and C418F) 8

Fig. S4 MD simulations comparing WT and M3 9

Fig. S5 Umbrella sampling analysis of WT and M3 10

Fig. S6 Optimization of Module 2 reaction conditions 10

Table S1. Kinetic parameters of enzymes involved in Module 1-2 11

Table S2. Information and characterisation of enzymes in Module 1-2 11

**Materials and methods**

**Materials**

The pET-28a(+) vector was obtained from Novagen (Beijing, China). *E. coli* BL21 (DE3) was purchased from Tiangen (Beijing, China). Restriction endonucleases, DNA ligase, and purification kits were sourced from Vazyme (Nanjing, China). Acetylacetone, 2,2’-azino-bis (3-ethylbenzothiazoline-6-sulfonic acid) (ABTS), pyruvate, L-lactate, methanol, formaldehyde, dihydroxyacetone (DHA), DHAP, serinol, ammonium sulfate, sodium formate, glucose, L-alanine, L-glutamate, L-aspartate, methylbenzylamine, isopropylamine, nicotinamide adenine dinucleotide (NAD^+^) and its reduced form (NADH) were purchased from Macklin Biochemical (Shanghai, China). Thiamine pyrophosphate (TPP), alcohol oxidase (A2404), horseradish peroxidase (HRP), and galactose oxidase (GalOD) were obtained from Sigma‒Aldrich (Shanghai, China). Catalase was purchased from SolarBio (Beijing, China). All other chemicals and reagents were of analytical grade.

**Expression and purification of recombinant proteins**

Some of the gene sequences were from our lab’s collection, while the remaining genes were synthesized by GenScript. All expression plasmids were derived from the pET-28a vector and transformed into *E. coli* BL21(DE3) competent cells. Sequencing-confirmed single clones were cultured in TB medium containing 50 μg/mL kanamycin at 37℃ with shaking at 180 rpm until the OD_600_ reached 0.6–0.8. Protein expression was induced with 1 mM IPTG, followed by incubation at 16℃ and 120 rpm for 24 h. Post-induction, the cells were harvested by centrifugation at 8,000 rpm for 10 min and resuspended in 50 mM phosphate buffer containing 300 mM NaCl. The crude enzyme solution was obtained from the supernatant after centrifugation at 12,000 rpm for 30 min. Proteins were purified using HisCap Co 6FF resin and eluted with an imidazole gradient. Desalting was performed with a 5 mL HisTrap HP column. The enzyme purity was assessed via SDS-PAGE, and the protein concentration was determined at 280 nm using a NanoDrop 2000 spectrophotometer.

**Measurement of enzyme activity and kinetic parameters**

For each enzyme system, a defined amount of the target enzyme was used to determine its activity and kinetic parameters. One unit (U) of enzyme activity is defined as the amount of enzyme required to produce 1 μmol of product per minute under the specified assay conditions. All measurements were performed in triplicate. Kinetic parameters (Vmax and *K*_m_) were calculated with GraphPad 8.0.

1. Methanol oxidase

The activity of methanol oxidase was assessed at 30℃ by measuring the increase in formaldehyde. Formaldehyde concentration was determined through a coupling reaction with acetylacetone and excess ammonium acetate, which resulted in a stable yellow product. The absorbance of the product was recorded at 414 nm. The assay mixture contained 100 mM HEPES buffer (pH 7.5), 20 mM acetylacetone, 50 mM acetic acid, 2 M ammonium acetate, and 20 mM methanol. For kinetic parameter determination, methanol concentrations varied from 1 to 150 mM.

1. Formolase

The activity of formolase was measured at 30℃ by quantifying the increase in DHA concentratio. DHA was oxidized by galactose oxidase (GalOD), producing hydrogen peroxide (H_2_O_2_). In the presence of horseradish peroxidase (HRP), ABTS was oxidized by H_2_O_2_ to form the ABTS cation radical (ABTS^•+^). The absorbance of ABTS^•+^ was monitored at 410 nm to quantify DHA production. The assay mixture consisted of buffer A (0.2 mg/mL GalOD, 24 U/mL HRP, 5 mM MgCl_2_, pH 7.5), buffer B (4 mM ABTS, 5 mM MgCl_2_, pH 7.5), and 10 mM formaldehyde in 100 mM HEPES buffer. For kinetic analysis, formaldehyde concentrations varied from 1 to 150 mM.

3.ω-Transaminases

The activity of ω-transaminases was assayed at 30℃ for 1 hour. The reaction mixture contained 100 mM HEPES buffer (pH 7.5), 5 mM MgCl_2_, 30 mM DHA, 150 mM L-Ala, and 0.5 mM PLP. For kinetic analysis, DHA concentrations ranged from 1 to 50 mM. After the reaction, the supernatant was collected by centrifugation and analyzed by high-performance liquid chromatography (HPLC) for activity determination.

4. L-lactate dehydrogenase

The activity of L-lactate dehydrogenase was assessed at 30℃ by monitoring the decrease in NADH absorbance at 340 nm. The assay mixture consisted of 100 mM HEPES buffer (pH 7.5), 5 mM MgCl_2_, 0.3 mM NADH, and 1 mM pyruvate. For kinetic parameter determination, pyruvate concentrations varied from 0.1 to 20 mM.

1. Alanine dehydrogenase

The activity of alanine dehydrogenase was assayed at 30℃ by measuring the decrease in NADH at 340 nm. The assay mixture contained 100 mM HEPES buffer (pH 7.5), 5 mM MgCl_2_, 0.3 mM NADH, and 1 mM pyruvate.

1. Formate Dehydrogenase

The activity of formate dehydrogenase was assayed at 30℃ by measuring the increase in NADH at 340 nm. The assay mixture contained 100 mM HEPES buffer (pH 7.5), 5 mM MgCl_2_, 0.3 mM NAD^+^, and 10 mM sodium formate. For kinetic parameter determination, sodium formate concentrations varied from 0.1 to 20 mM.

1. Glucose dehydrogenase

The activity of glucose dehydrogenase was assayed at 30℃ by measuring the increase in NADH at 340 nm. The assay mixture contained 100 mM HEPES buffer (pH 7.5), 50 mM glucose, 5 mM NAD^+^, and 5 mM MgCl_2_.

**Classical molecular dynamics simulations**

The initial protein structure was optimized using 2,500 steps of steepest descent followed by 2,500 steps of conjugate gradient minimization with the Amber22 software suite [1]. Protein residues were parameterized with the AMBER14SB force field [2], while DHA and PMP were parameterized with the GAFF force field [3]. The system was solvated in a periodic dodecahedron with a 10 Å buffer between the solute and the box edges. To neutralize the system, appropriate amounts of Na^+^ and Cl^-^ ions were added. The system was first equilibrated in a 2 ns NVT heating simulation, during which the temperature was gradually increased to 300 K using the Langevin thermostat. A time step of 2.0 fs was applied, with hydrogen bonds constrained using the SHAKE algorithm. Following heating, a 2 ns NPT simulation was carried out to further relax the system, maintaining the temperature at 300 K with the Langevin thermostat, a 2.0 fs time step, and SHAKE constraints on hydrogen bonds. Electrostatic interactions were computed using the particle mesh Ewald method, with a non-bonded interaction cutoff of 10 Å. Production MD simulations were performed for 200 ns with a 2 fs time step, saving coordinates every 2 ps. The resulting trajectories were analyzed using the cpptraj tool.

**Supporting Figures**


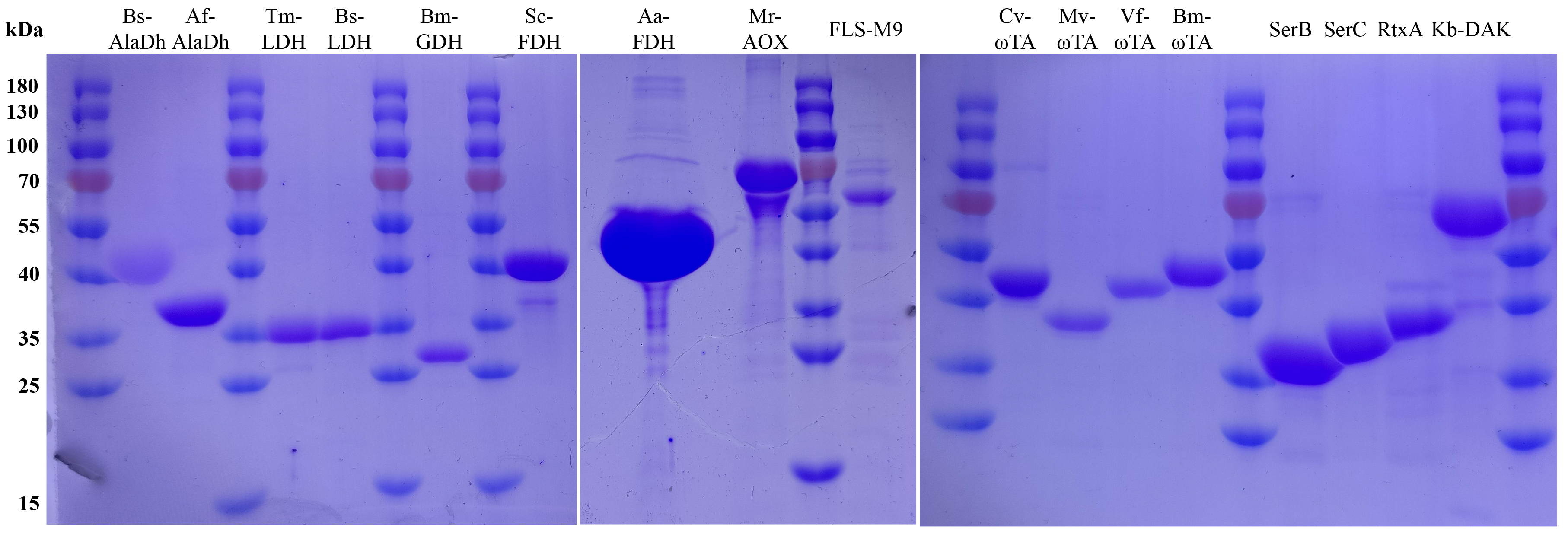


**Fig. S1.** SDS-PAGE analysis of recombinant enzymes involved in the methanol-to-serinol cascade.


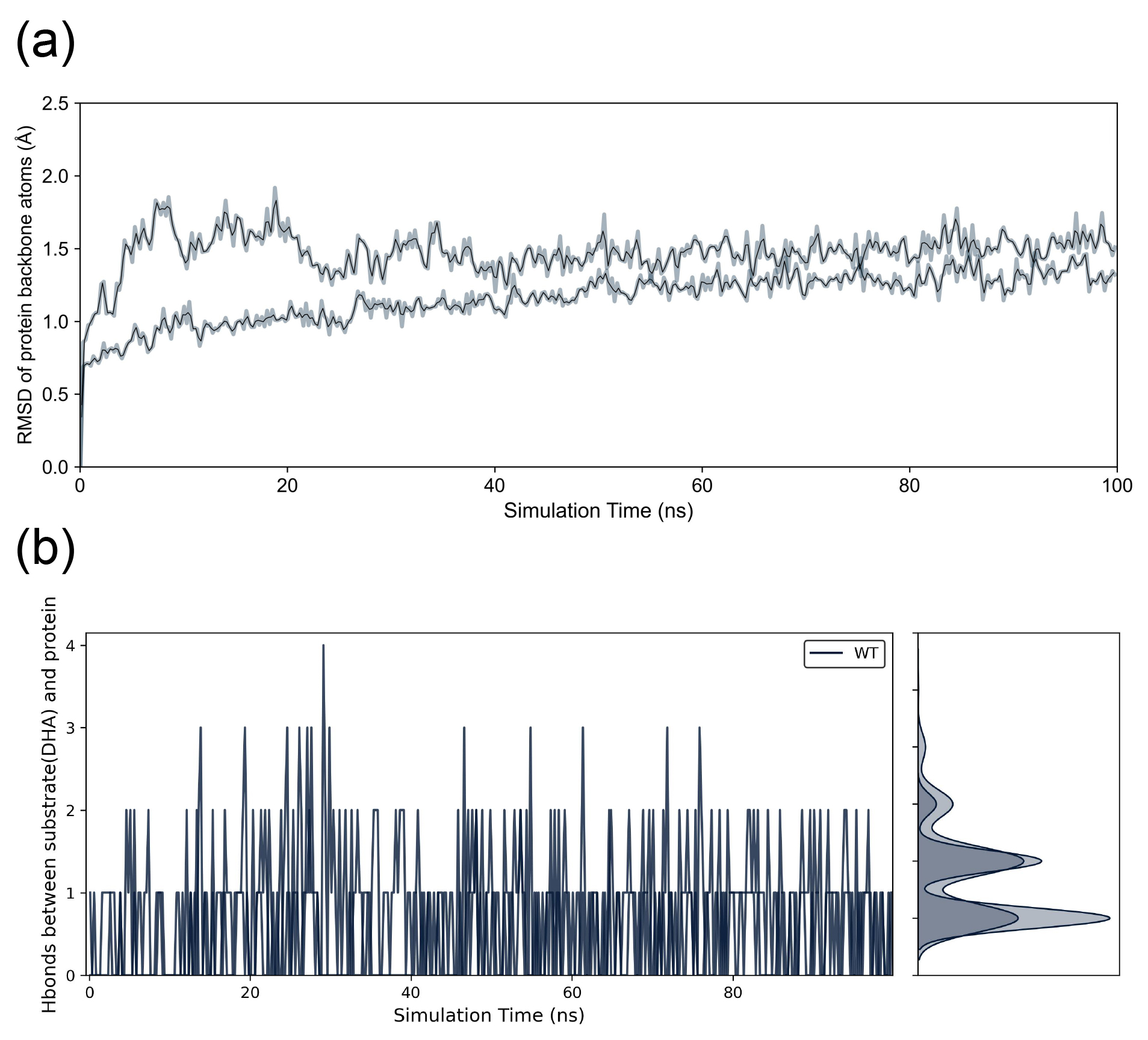


**Fig. S2**. MD simulations of Cv-ωTA (WT) with DHA. (a) RMSD of protein backbone atoms during a 100 ns simulation. (b) Number of hydrogen bonds formed between DHA and active-site residues over time.


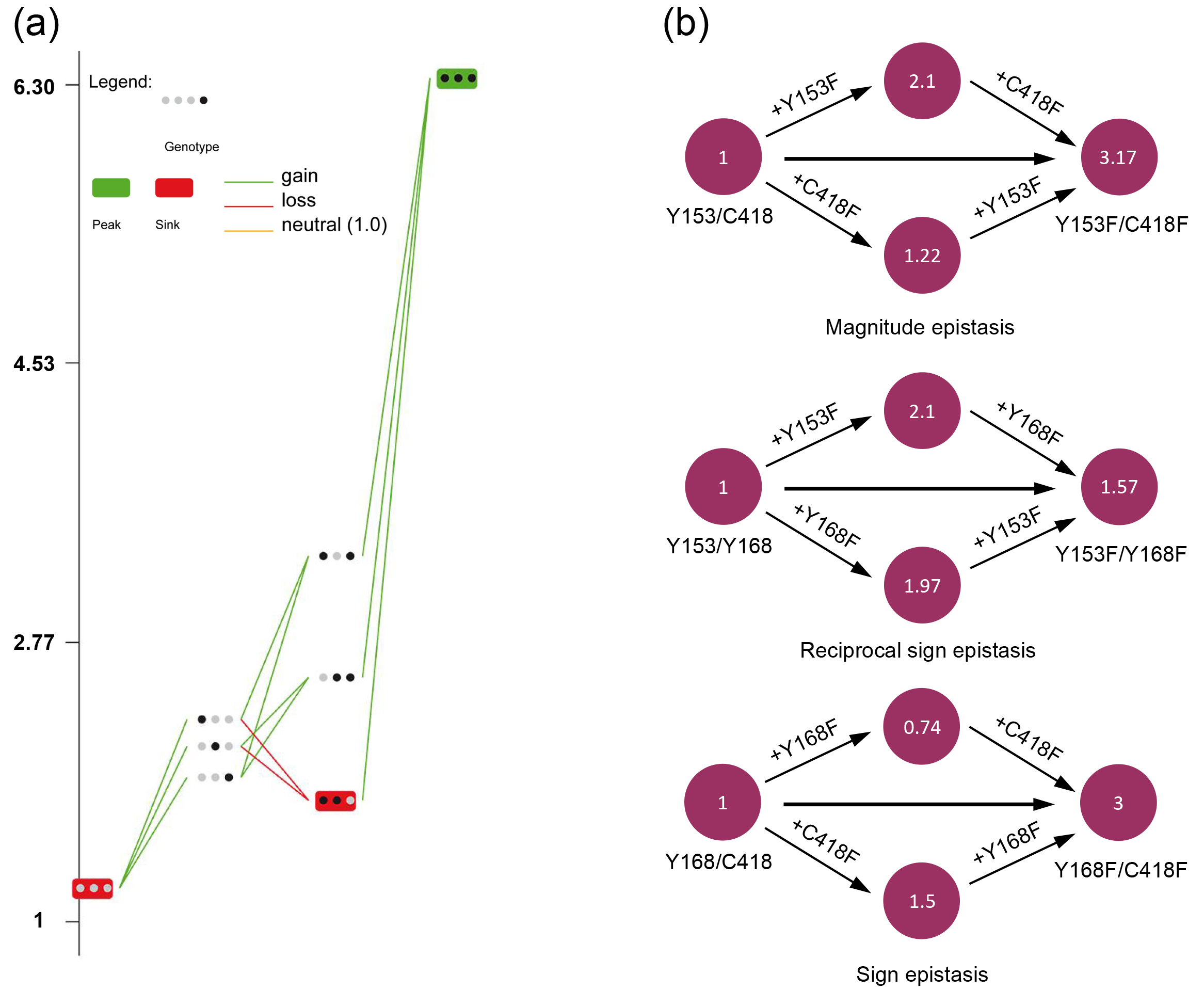


**Fig. S3**. Epistasis analysis of key Cv-ωTA mutations (Y153F, Y168F, and C418F). (a) Evolutionary trajectories of Cv-ωTA variants analyzed with MAGELLAN, showing mutational paths with green and red lines. (b) Epistatic interactions among Y153F, Y168F, and C418F mutations, including magnitude epistasis (top), reciprocal sign epistasis (middle), and sign epistasis (bottom).


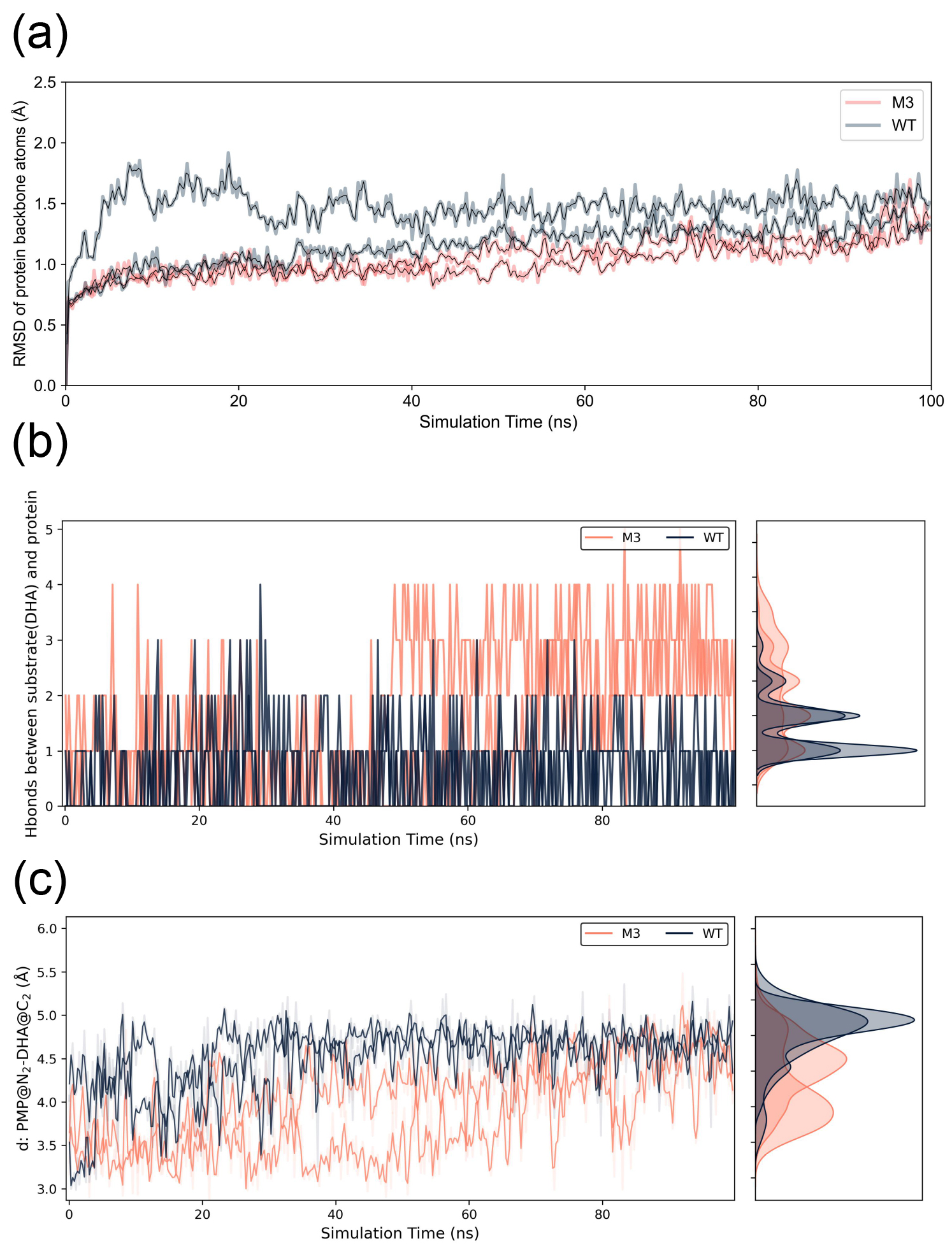


**Fig. S4**. MD simulations comparing WT and M3. (a) RMSD of protein backbone atoms during a 100 ns simulation. (b) Number of hydrogen bonds formed between DHA and active-site residues. (c) Distance between PMP (N_2_) and the DHA carbonyl carbon (C_2_) during simulation.


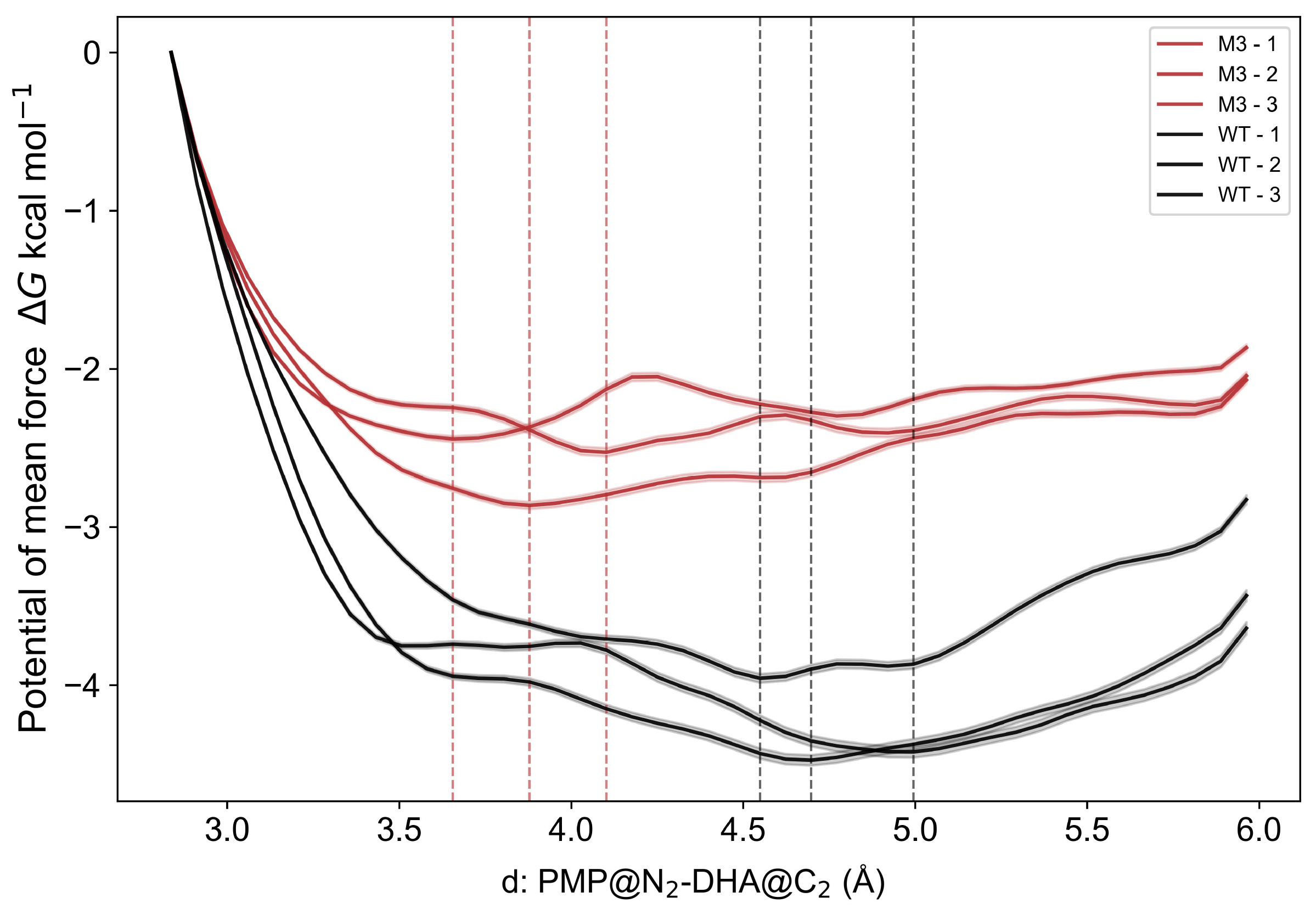


**Fig. S5**. Umbrella sampling analysis of WT and M3. Potential of mean force (PMF) profiles of WT and M3 along the reaction coordinate defined by the distance between PMP–NH₂ and the DHA carbonyl carbon.


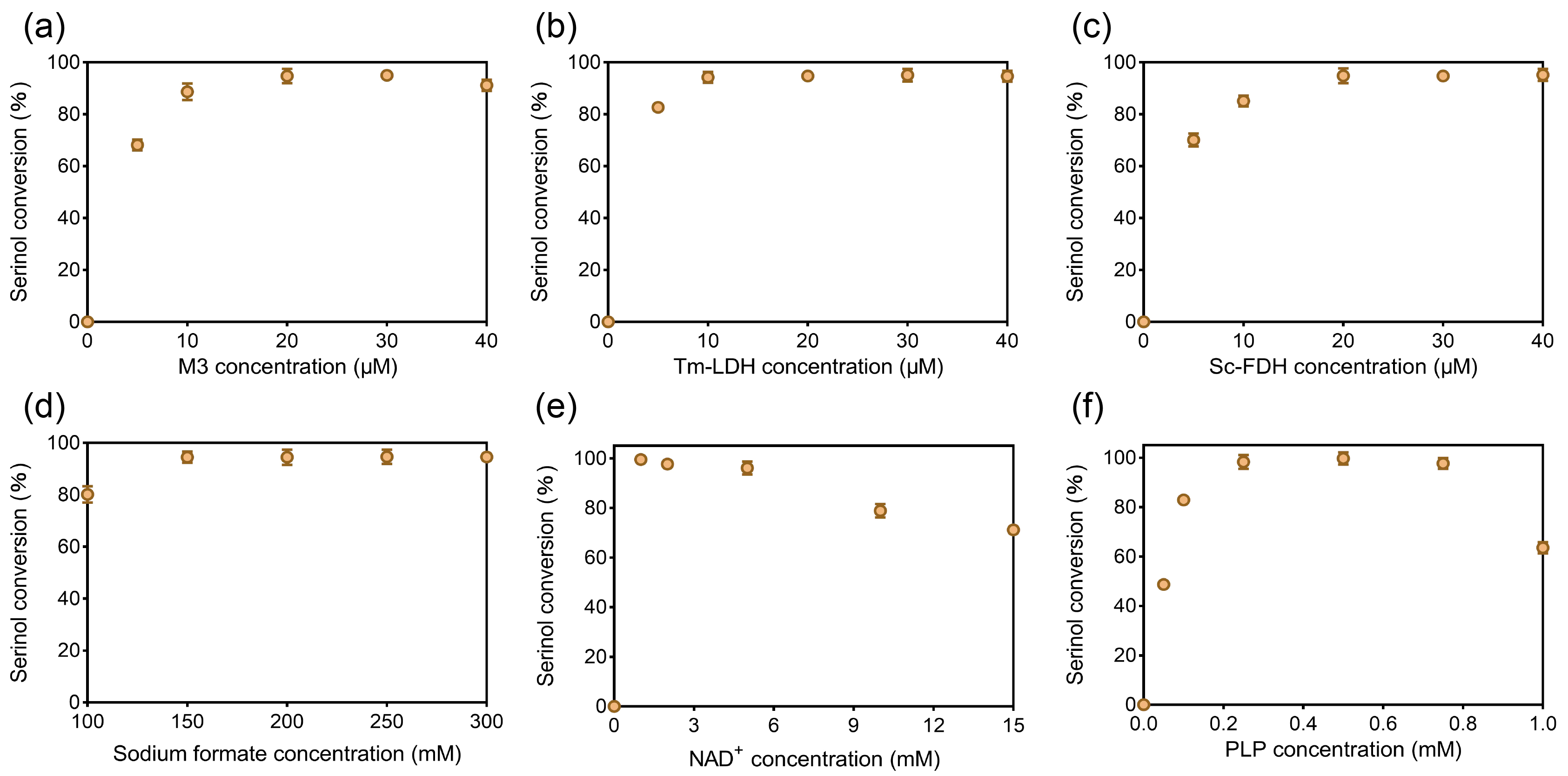


**Fig. S6**. Optimization of Module 2 reaction conditions. Conditions were optimized for (a) M3 concentration (0–40 μM), (b) Tm-LDH concentration (0–40 μM), (c) Sc-FDH concentration (0–40 μM), (d) sodium formate concentration (100–300 mM), (e) NAD⁺ concentration (0–15 mM), and (f) PLP concentration (0–1.0 mM).

**Supporting Tables**

**Table S1. Kinetic parameters of enzymes involved in Module 1-2.**

| **Abbreviation** | ***kcat*(s^−1^)** | ***K*m (mM)** | ***kcat*/*K*m (mM^−1^ s^−1^)** |
| --- | --- | --- | --- |
| Cv-ωTA | 1.61 ± 0.07 | 15.2 ± 0.11 | 0.10 |
| M3 | 3.04 ± 0.11 | 7.22 ± 0.13 | 0.42 |

**Table S2**. Information and characterization of enzymes in Module 1-2

| **Enzyme** | **Abbreviation** | **Sources** | **GenBank ID** | **Specific activity (U/mg)** |
| --- | --- | --- | --- | --- |
| Methanol oxidase | Mr-AOX | *Moniliophthora roreri* | ESK84078 | 3.50 ± 0.17 |
| Formolase | FLS-M9 | *Pseudomonas fluorescens* | - | 1.59 ± 0.35 |
| ω-Transaminases | Cv-ωTA | *Chromobacterium violaceum* | WP_011135573.1 | 0.08 ± 0.01 |
| L-lactate dehydrogenase | Bs-LDH | *Bacillus subtilis* | NWN95529.1 | 28.91 ± 1.30 |
|  | Tm-LDH | *Thermotoga maritima* | WP_004082418.1 | 50.48 ± 3.72 |
| Alanine dehydrogenase | Af-AlaDH | *Archaeoglobus fulgidus* | WP_010879161.1 | 17.20 ± 0.76 |
|  | Bs-AlaDH | *Bacillus subtilis* | WP_003243280.1 | 11.90 ± 0.54 |
| Formate dehydrogenase | Aa-FDH | *Ancylobacter aquaticus* | BAC65346.1 | 0.65 ± 0.03 |
|  | Sc-FDH | *Saccharomyces cerevisiae* | CAI4824870.1 | 5.09 ± 0.12 |
| Glucose dehydrogenase | Bm-GDH | *Bacillus megaterium* | - | 90.00 ± 2.28 |

References

1. Case DA, Aktulga HM, Belfon K, Cerutti DS, Cisneros GA, Cruzeiro VW, et al. The AmberTools. J Chem Inf Model 2023;63(20):6183–6191. <https://doi.org/10.1021/acs.jcim.3c01153>

Maier JA, Martinez C, Kasavajhala K, Wickstrom L, Hauser KE, Simmerling C. ff14SB: improving the accuracy of protein side chain and backbone parameters from ff99SB. J Chem Theory Comput 2015;11(8):3696–3713. <https://doi.org/10.1021/acs.jctc.5b00255>

(3) Sprenger KG, Jaeger VW, Pfaendtner J. The general AMBER force field (GAFF) can accurately predict thermodynamic and transport properties of many ionic liquids. J Phys Chem B 2015;119(18):5882–5895. https://doi.org/10.1021/acs.jpcb.5b00689
